# Le syndrome de Cushing paranéoplasique chez le jeune adulte, une entité rare avec un réel challenge diagnostic: à propos d’un cas

**DOI:** 10.11604/pamj.2025.51.47.46381

**Published:** 2025-06-17

**Authors:** Ghizlane Sabbar, Kaoutar Rifai, Fatima Toulali, Hinde Iraqi, Mohamed El Hassan Gharbi

**Affiliations:** 1 Service d'Endocrinologie et Maladies Métaboliques, Centre Hospitalier Universitaire Ibn Sina, Rabat, Maroc,; 2 Faculté de Médecine et de Pharmacie Rabat, Université Mohammed V Souissi, Avenue Belarbi El Alaoui, BP 6203, Rabat, Maroc

**Keywords:** Syndrome de Cushing paranéoplasique, tumeurs neuroendocrines, anticortisoliques, surrenalectomie bilatérale, cas clinique, Paraneoplastic Cushing's syndrome, neuroendocrine tumors, steroidogenesis inhibitors, bilateral adrenalectomy, case report

## Abstract

Le syndrome de Cushing paranéoplasique est une cause rare du syndrome de Cushing ACTH-dépendant, rarement observée chez le jeune adulte, et pose souvent le problème de diagnostic étiologique. Nous rapportons le cas d'une jeune patiente de 16 ans, qui a présenté un tableau d'hypercorticisme sévère sans diagnostic étiologique initialement, d'où l'aggravation de son tableau clinique menant à la réalisation d'une surrenalectomie bilatérale de sauvetage. La pathologie tumorale en cause a été diagnostiquée plusieurs mois après grâce à l'imagerie fonctionnelle. La patiente a bénéficié d'une exérèse chirurgicale avec une nette amélioration. Notre cas illustre à la fois une forme rare du syndrome de Cushing ACTH dépendant, ainsi que l'âge très jeune de sa survenue, et montre la difficulté d'établir un diagnostic étiologique précis, ce qui nécessite souvent l'utilisation de technique d'imagerie plus poussée.

## Abstract (en)

Paraneoplastic Cushing's syndrome is a rare cause of ACTH-dependent Cushing's syndrome, rarely observed in young adults, and often presents a challenge in determining its etiology. We report the case of a 16-year-old female patient presenting with a severe hypercortisolism, for which no underlying cause could initially be identified. Her clinical condition worsened, leading to emergency bilateral adrenalectomy as a life-saving measure. The underlying tumor pathology was diagnosed several months later on the basis of functional imaging. The patient underwent surgical excision, resulting in significant clinical improvement. This case highlights both a rare form of ACTH-dependent Cushing's syndrome and the very young age of onset, demonstrating the difficulty in establishing a precise etiological diagnosis, often requiring advanced imaging techniques.

## Introduction

Le syndrome de Cushing paranéoplasique est une situation rare, secondaire à des tumeurs neuroendocrines qui se développent dans de nombreux organes, principalement au niveau des poumons [1]. La tumeur en cause peut ne pas se révéler au début ce qui pose le problème de diagnostic étiologique et des défis thérapeutiques, comme dans le cas de notre patiente.

## Patient et observation

**Présentation du patient:** nous rapportons le cas d'une jeune patiente de 16 ans, sans antécédent particulier, qui a été adressée dans notre formation pour la suspicion d'un syndrome de Cushing devant une prise de poids importante et rapide (10kg en trois mois) et une érythrose faciale.

**Résultats cliniques:** l'évaluation clinique a montré une d'obésité facio-tronculaire avec un abdomen en tablier et un indice de masse corporelle à 35kg/m^2^, des vergetures pourpres et larges sur le ventre et la face interne des cuisses, une atrophie cutanée, avec une mélanodermie franche, sans signes de virilisation.

**Démarche diagnostique:** devant ce tableau clinique d'un syndrome de Cushing, on a réalisé un dosage de cortisol de minuit qui est élevé à 100ng/ml, un CLU (cortisol libre urinaire) de 24 heures qui est positif: supérieur à 5 fois la normale, ainsi que le dosage de l'ACTH qui est élevé à 31 pg/ml. On a conclu à un syndrome de Cushing ACTH dépendant et on a complété par L'IRM (imagerie par résonnance magnétique) hypothalamo-hypophysaire. Cette dernière était en faveur de deux adénomes hypophysaires infra-centimétriques bilatéraux. Le diagnostic de la maladie de Cushing a été retenu, et on a réalisé chez la patiente une adénomectomie par voie transphénoïdale. L'étude anatomopathologique n'a pas révélé d'anomalie, et l'évolution a été marquée par la persistance d'un hypercorticisme clinico-biologique. D'où la reprise de la patiente et la réalisation d'une résection totale de l'hypophyse (après un staff multidisciplinaire, et consentement des parents). Et pour la deuxième fois, l'étude anatomo-pathologique avec un complément par immunohistochimie n'a pas révélé d'anomalie sur la pièce opératoire.

**Figure 1:**
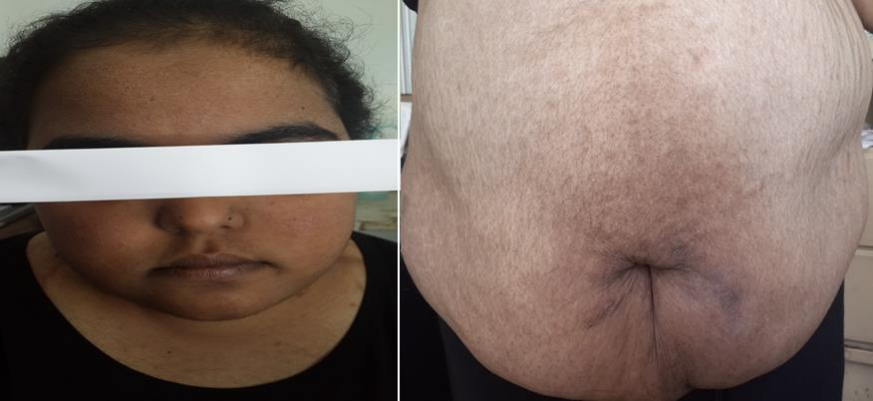
obésité facio-tronculaire avec une mélanodermie intense chez notre patiente

**Figure 2:**
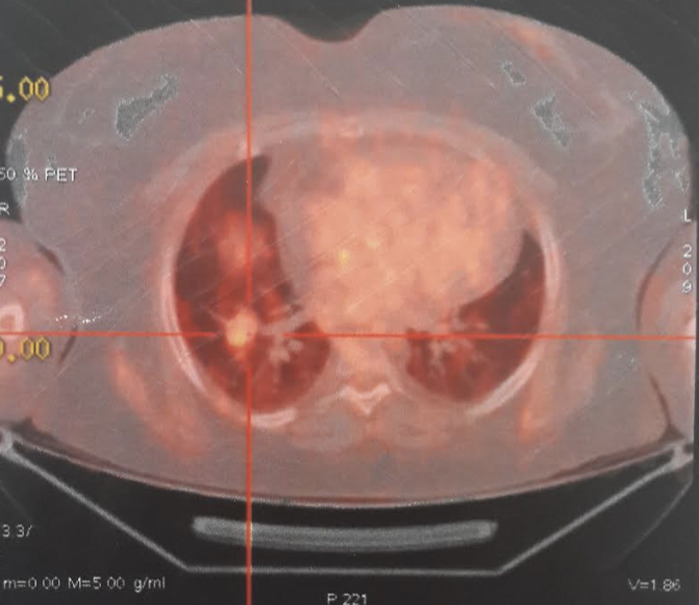
foyer pulmonaire nodulaire actif pathologique sur le 18 FDG PET, de localisation lobaire inférieure droite

**Chronologie:** l'évolution a été marquée par l'aggravation du tableau clinique qui devient sévère et agressif, avec une accentuation de la mélanodermie (Figure 1) et l'installation d'une hypertension artérielle secondaire ainsi que des infections sévères des parties molles. Sur le plan biologique, on a eu une persistance d'un CLU élevé supérieur à 5 fois la normale, une élévation de l'ACTH à 130 pg/ml, l'apparition d'une hypokaliémie sévère à 2,5 meq/l, avec un diabète (HbA1c: 10%) et une cytolyse hépatique qui dépasse 5 fois la normale, malgré un traitement anticortisolique à base de kétoconazol 600mg/jour et métyrapone 3g/jour. Les nouvelles explorations radiologiques faites de radiographie de poumon, IRM hypothalamo-hypophysaire, TDM TAP (tomodensitométrie thoraco-abdomino-pelvienne), ainsi que le Pet scan à la 18 FDG (Tomographie par émission de positons à la 18 fluorodésoxy-glucose) n'ont pas révélé d'anomalie. Devant cette situation délicate et la mise en jeu du pronostic vital chez notre patiente, une surrenalectomie bilatérale de sauvetage a été réalisée, et la patiente a été mise sous hydrocortisone 30mg/jour et fludrocortisone 0,1mg/jour. La ré-exploration ultérieure (trois mois plus tard) par TEP scan à la 18 FDG a révélé deux foyers pulmonaires nodulaires lobaires inférieurs droits actifs pathologiques (Figure 2). Ainsi le diagnostic d'un syndrome de Cushing paranéoplasique secondaire à une tumeur bronchique a été retenu.

**Intervention thérapeutique:** la patiente a bénéficié d'une lobectomie droite. L'étude anatomopathologue de la pièce opératoire a montré une localisation pulmonaire de tumeurs carcinoïdes typiques mesurant 2.5 et 1.5cm, de bas grade ne nécessitant pas de thérapie complémentaire.

**Suivi et résultats des interventions thérapeutiques:** l'évolution chez notre patiente a été marquée par une nette amélioration clinique, faite d'une régression de la mélanodermie avec une perte de poids (5kg en 2 mois), la guérison de son HTA et de son diabète, et la normalisation de tous les paramètres biologiques: le CLU, l'ACTH, la kaliémie et le bilan hépatique.

**Perspective de la patiente:** cette évolution spectaculaire, a permis à notre patiente de reprendre sa scolarité après quelques mois de convalescence, de suivi et de soutien psychologique. Elle nous exprime, à chaque contrôle dans notre formation, son soulagement et sa satisfaction après ce long combat.

**Consentement éclairé:** la patiente a déclaré son consentement librement et de façon éclairée, afin de permettre la réalisation et la publication de ce manuscrit.

## Discussion

Notre cas illustre une forme rare aussi bien par rapport au syndrome de Cushing paranéoplasique, qu'à l'âge jeune de notre patiente. Cette forme représente 10 à 17% des cas de syndrome de Cushing ACTH dépendants [2,3]. Selon les données de la littérature, L'âge moyen de sa survenue est de 53 ans (26-76 ans), orientant vers une prédominance de ce syndrome chez la population adulte. Les cas rapportés chez le sujet jeune comme notre patiente, sont rares, avec deux cas publiés âgés de 9 et 18 ans [4]. Les symptômes sont secondaires à la sécrétion d'ACTH par les cellules malignes, et à l'effet des anticorps des cellules néoplasiques sur les cellules saines [3-6]. Il s'agit d'un tableau d'hypercorticisme sévère avec une mélanodermie franche, qui évolue vers une altération de l'état général, ce qui est le cas chez notre patiente [7].

Le diagnostic est confirmé par la rupture du rythme circadien de sécrétion de cortisol, l'augmentation du cortisol salivaire et du cortisol libre urinaire de 24H (CLU) qui dépasse deux fois la normale, avec un taux d'ACTH très élevé, non freinable par la dexaméthasone à forte dose [8]. Cette sécrétion importante d'ACTH est secondaire à des tumeurs neuroendocrines de taille et de localisation variables dont le diagnostic positif pose un réel challenge. C'est un type de tumeur relativement rare et hétérogène, représentant environ 2% de toutes les tumeurs malignes [3]. Il s'agit de tumeurs bronchiques et pulmonaires dans plus de 50% des cas, viennent en second lieu les tumeurs thymiques, puis les carcinomes pancréatiques, les cancers médullaires de la thyroïde et les phéochromocytomes [7].

Dans le cas de notre patiente il s'agit de deux tumeurs carcinoïdes typiques de localisation pulmonaire de bas grade. C'est des tumeurs neuroendocrines à malignité réduite, représentant 1 à 2% de toutes les tumeurs malignes pulmonaires chez l'adulte, et 20 à 30% de toutes les tumeurs carcinoïdes [4]. Elles peuvent être responsables de signes respiratoires: toux chronique, dyspnée, hémoptysies, comme elles peuvent être asymptomatiques dans 20 à 50% des cas, comme chez notre patiente qui n'avait aucun signe respiratoire. L'association de ces tumeurs avec le syndrome de Cushing, ce qui est rare (1-2% de cas), constitue un facteur d'agressivité [4].

Le diagnostic de localisation de ces tumeurs est souvent tardif, avec un délai moyen pouvant aller jusqu'à 24 mois. L'imagerie conventionnelle utilisée en première intention, à savoir la tomodensitométrie et l'IRM, n'a pas la sensibilité et la spécificité requises pour identifier ces lésions ectopiques [4]. Pour cela on a souvent recours, en deuxième intention, à l'imagerie fonctionnelle qui permet de détecter environ 80% des tumeurs non identifiées. Il s'agit du TEP aux peptides 68Ga-DOTA qui offre la meilleure sensibilité, suivi par le TEP à la 18F-fluorodéoxyglucose (FDG) qui a été effectué chez notre patiente, puis la scintigraphie à l'Octréotide 5 (111 In octreotid) et à la MIBG-123I [9]. A noter que chez 20% des patients, la tumeur reste non détectable pendant plusieurs années [2-5].

Le traitement du syndrome de Cushing paranéoplasique repose sur l'exérèse chirurgicale de la tumeur responsable de la sécrétion d'ACTH, ce qui est curatif chez > 80% des patients [4-7]. Mais, en parallèle aux procédures diagnostiques qui risquent de prendre du temps, un traitement médical à base d'inhibiteurs de la stéroïdogenèse à effet rapide, en monothérapie ou en association (kétoconazol, métyrapone, l'osilodrostat, pasiréotide), doit être initié: cela permettra d'atténuer la sévérité du tableau clinique et biologique, et de stabiliser le patient en attendant d'avoir un diagnostic clair. Sans oublier la prise en charge immédiate des comorbidités et des complications [10].

Dans le protocole thérapeutique, la surrénalectomie bilatérale doit être proposée en dernière ligne, en cas d'indisponibilité de médicaments anticortisoliques, d'échec d'un traitement médical anticortisolique bien conduit, d'intolérance médicamenteuse ou en cas de contexte néoplasique menaçant. On l'a réalisé chez notre jeune patiente, vue la mise en jeu du pronostic vital malgré un traitement médical bien conduit [1].

## Conclusion

Notre observation expose une forme rare du syndrome de Cushing ACTH-dépendant, avec les défis diagnostiques rencontrés en pratique clinique devant ce syndrome. Le recours à l'imagerie fonctionnelle est d'un grand intérêt dans le bilan de localisation des tumeurs neuroendocrines en cause, avec la nécessité dans certaine situation, comme celle de notre patiente, de le refaire dans un délai de quelques mois en cas d'un bilan initial négatif. Ces tumeurs sont dans la majorité à malignité réduite, mais exposent le patient à un hypercorticisme sévère pouvant mettre en jeu le pronostic vital.
